# Comment on Abbas et al. The Safety and Efficacy of Nusinersen in the Treatment of Spinal Muscular Atrophy: A Systematic Review and Meta-Analysis of Randomized Controlled Trials. *Medicina* 2022, *58*, 213

**DOI:** 10.3390/medicina58060766

**Published:** 2022-06-06

**Authors:** Ahmed Aljabali, Maha Abdo, Ahmed Negida

**Affiliations:** 1 Neuroscience Department, Medical Research Group of Egypt, Zagazig 44523, Egypt; aljbalia37@gmail.com (A.A.); dr.mahha92@gmail.com (M.A.); 2 Faculty of Medicine, Jordan University of Science & Technology, Irbid 22110, Jordan; 3 Arab Council for Health Specializations, Sana’a 11274, Yemen; 4 Faculty of Medicine, Zagazig University, Zagazig 44519, Egypt; 5 School of Pharmacy and Biomedical Sciences, University of Portsmouth, Portsmouth PO1 2DT, UK

We read with interest the article by Abbas et al. [1] which reported a meta-analysis of randomized controlled trials to evaluate the safety and efficacy of nusinersen in treating patients with spinal muscular atrophy (SMA). Although the topic is interesting and important for clinical practice, we found some methodological and statistical issues in the article that are worth acknowledging.

In their analysis (Figure 3A,B), the authors pooled data on the motor improvement of patients with different types of SMA; in Acsadi et al. [2], the study included patients with both infantile-onset (type 1 SMA) and later-onset SMA (type 3 and 4 SMA) with no data for SMA type 2 and no separate data for each SMA type reported in the trial. The authors pooled these data with Finkel et al. [3] who only evaluated SMA patients with type 1. Furthermore, the authors mistakenly reversed the forest plot labels for the left and right directions.

Similarly, in Figure 4, the authors pooled data about the nusinersen safety for patients with different types of SMA without distinction. For example, Acsadi et al. [2] studied SMA types 1, 3, and 4, Mercuri et al. [4] studied SMA type 2, and Finkel et al. [3] studied SMA type 1.

Throughout the entire article, the authors acknowledge that the nusinersen is safe and effective for SMA treatment. However, it should be clarified that the results and conclusions do not generalize to all SMA patients as follows:

1. Evidence on its efficacy of nusinersen in some SMA subgroups is lacking and inconclusive. For example, no evidence was provided support the use of nusinersen for patients with respiratory insufficiency (those who need invasive or noninvasive ventilation for more than 6 hours per day), bulbar manifestations, gastric feeding tube, severe contractures or severe scoliosis, or medical disability. Clinical trials usually exclude those patient subgroups, and it is unclear whether those patients with severe disease associated with respiratory insufficiency can benefit from this drug.
2. Adult SMA patients were not included in this meta-analysis; the same results and conclusions do not apply to adult SMA. Clinical trials assessed the efficacy of nusinersen within a 1–2-year period. There is no evidence to support the long-term efficacy and safety of nusinersen.
3. Given the high costs of nusinersen treatment, proper patient selection is important to optimize the cost-effectiveness ratio and to ensure appropriate resource allocation. Added to that, onasemnogene abeparvovec-xioi (Zolgensma) and risdiplam (Evrysdi) are two other disease-modifying therapies for SMA that have shown to be safe and effective and were approved by the FDA [5,6,7]. These treatments are now part of the SMA standard of care. For example, for SMA patients with severe scoliosis and difficult intrathecal access, the nusinersen treatment might not be suitable; alternatively, risdiplam has the advantage of an oral route of administration.

Based on existing evidence, the UK’s National Institute for Health and Care Excellence (NICE) guidelines employs a set of criteria to select SMA patients for nusinersen [8] (Table 1).

In conclusion, the present evidence supports the short-term efficacy and safety of nusinersen in children with SMA types 1, 2, and 3 who fall within the selection criteria of the published trials. For SMA children who do not meet the selection criteria and adult SMA, the benefits and risks of nusinersen are uncertain. The quality of current evidence is relatively low due to the small number of available trials, which could be justified by SMA being a rare disease. Future clinical trials should consider [1] broader selection criteria, [2] longer-term follow-up, and [3] potential treatment comparisons, switches, and combinations with the two other disease-modifying therapies.

## Figures and Tables

**Table 1:** Shows the eligibility criteria for nusinersen treatment—adopted from UK’s NICE guidelines [8].

*Patients with early- (type 1) or later-onset (types 2 and 3) SMA and people with presymptomatic SMA with homozygous gene deletion or homozygous mutation or compound heterozygous mutation of the SMN1 gene (chromosome 5) found via presymptomatic genetic testing* *Must not have type 4 SMA; that is, must not have symptom onset at or after 19 years of age* *Must not have type 0 SMA* *The intrathecal injection must be technically feasible in the opinion of the treating clinician and not contraindicated* *No permanent ventilation (16 or more hours per day for 21 consecutive days in the absence of acute reversible infection) or tracheostomy requirement at baseline* *Must not have had spinal fusion surgery after a diagnosis of scoliosis that, in the opinion of the treating clinician, prohibits safe administration of nusinersen* *Must not have severe contractures that, in the opinion of the treating clinician, prohibit measurement of motor milestones* *Patients must still be independently ambulant if independent ambulation is gained before starting therapy, except for pediatric patients who have lost independent ambulation in the previous 12 months.”*
